# Complete mitochondrial genomes of *Callospermophilus lateralis* and *Urocitellus richardsonii* (Rodentia: Sciuridae)

**DOI:** 10.1080/23802359.2016.1168716

**Published:** 2016-06-20

**Authors:** Leping Zhang, Yuanyuan Huang, Kenneth B Storey, Danna Yu, Jia-Yong Zhang

**Affiliations:** a College of Chemistry and Life Science, Zhejiang Normal University, Jinhua, Zhejiang Province, China;; b Department of Biology, Carleton University, Ottawa, Ontario, Canada;; c Key Lab of Wildlife Biotechnology, Conservation and Utilization of Zhejiang Province, Zhejiang Normal University, Jinhua, Zhejiang Province, China

**Keywords:** Golden-mantled ground squirrel, mitochondrial genome, Richardson’s ground squirrel, Sciuridae

## Abstract

The complete mitochondrial genomes of *Callospermophilus lateralis* and *Urocitellus richardsonii* (Rodentia: Sciuridae) were sequenced to analyze the gene arrangement and discuss the phylogenetic relationship of species within the Xerinae. The genomes are circular molecules of 16,457 bp and 16,460 bp in length, respectively, including the 37 genes typically found in other squirrels. The AT content of the overall base composition is 63.2% for both species. The length of control region for *C. lateralis* is 1009 bp with 62.8% AT content; the corresponding values for *U. richardsonii* are 1012 bp and 62.0% AT content. In BI and ML phylogenetic trees, the monophyly of the family Sciuridae and subfamilies Callosciurinae and Sciurinae are well supported, but the monophyly of Xerinae is not supported. Within the Xerinae, the relationship of (*Tamias sibiricus* + (*Callospermophilus lateralis +* (*Marmota himalayana* + (*Urocitellus richardsonii* + (*Ictidomys tridecemlineatus* + (*Cynomys leucurus* +* Cynomys ludovicianus*)))))) is well supported. However, *Tamiops swinhoei* (Xerinae) within the subfamily Callosciurinae is a clade sister to *Dremomys rufigenis* (Callosciurinae). *Spermophilus dauricus* (Xerinae) within subfamily Sciurinae is a sister clade to *Sciurus vulgaris* (Sciurinae). The monophyly of Xerinae is failed to support in this study.

Ground squirrels (*Spermophilus*), prairie dogs (*Cynomys*) and their relatives are among the most intensively studied groups of mammals with respect to their ecology and behavior (Harrison et al. 2003). The phylogenetic relationship of the family Sciuridae has been revised and discussed based on molecular markers (Harrison et al. 2003; Herron et al. 2004; Helgen et al. 2009). We determined the sequences of the complete mitochondrial genomes of *Callospermophilus lateralis* (=*Spermophilus lateralis*) and *Urocitellus richardsonii* (=*Spermophilus richardsonii*) (Rodentia: Sciuridae) to analyze the gene arrangement and to further explore the phylogenetic relationship of species within the Xerinae. Both species are well-studied models of mammalian hibernation.

Golden-mantled ground squirrels (*C. lateralis*) were collected in the Crooked Creek area of the White Mountains of California, CA, whereas Richardson’s ground squirrels (*U. richardsonii*) were collected near Calgary, Alberta, Canada. The tissues samples (JBHS2015 and DSS2015) are stored and archived at the Biology Department, Carleton University, Canada. Liver samples from both species were sampled, flash frozen in liquid nitrogen and held at −80 °C until use. Whole genomic DNA was extracted from a liver sample of each species. The DNA samples of *C. lateralis* (JBHS2015) and *U. richardsonii* (DSS2015) were stored at College of Chemistry and Life Science, Zhejiang Normal University, China. DNA fragments were amplified using 10 pairs of highly conserved primers which were designed according to the method of Li et al. (2015) and Zhang et al. (2015). DNA was amplified by PCR and both strands were sequenced.

*Callospermophilus lateralis* and *U. richardsonii* showed the typical rodent-type arrangement of their mitocondrial genomes, being circular molecules of 16,457 bp and 16,460 bp in length, respectively. Each encoded the 37 genes typically found in other squirrel species. The AT content of the overall base composition is 63.2% for both species. The length of control region of *C. lateralis* is 1009 bp with 62.8% AT content whereas the length of control region of *U. richardsonii* is 1012 bp with 62.0% AT content. Bayesian inference (BI) and maximum-likelihood (ML) trees were constructed using the 13 protein-coding genes (PCGs) from 20 species including *Castor fiber*, *C. canadensis* (Castoridae) and *Myoxus glis* (Gliridae) as outgroups to confirm the phylogenetic relationship among the Sciuridae (Figure 1). To select conserved regions of the nucleotide, each alignment was performed by Gblock 0.91b (Castresana 2000) using default settings. BI analysis and ML analysis was performed by MrBayes3.1.2 (Huelsenbeck & Ronquist 2001) and PAUP*4.0b10 (Swofford 2002), respectively. In BI and ML phylogenetic trees, the monophyly of the family Sciuridae and the subfamilies Callosciurinae and Sciurinae are well supported (1.00 in BI, 100% in ML), but the monophyly of Xerinae failed. Within the Xerinae, the relationship of (*Tamias sibiricus* + (*Callospermophilus lateralis +* (*Marmota himalayana* + (*Urocitellus richardsonii* + (*Ictidomys tridecemli-neatus* + (*Cynomys leucurus* +* Cynomys ludovicianus*))))))) is also well supported (1.00 in BI and 100% in ML). However, *Tamiops swinhoei* (Xerinae) within the subfamily Callosciurinae is a clade sister to *Dremomys rufigenis* (Callosciurinae) and *Spermophilus dauricus* (Xerinae) within the subfamily Sciurinae is a sister clade to *Sciurus vulgaris* (Sciurinae). The monophyly of Xerinae is failed to support in this study. Overall, the sequences of more mitochondrial genomes from Sciruidae species are needed in order to fully assess the monophyly of Xerinae.

**Figure 1.**
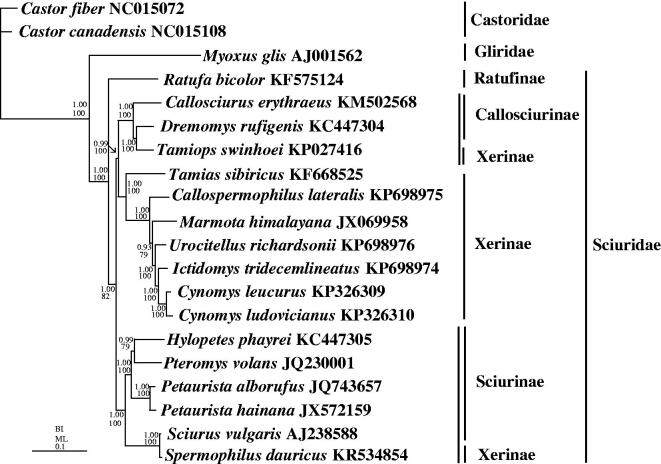
Phylogenetic tree of the relationships among 20 species of Rodentia based on the nucleotide dataset of the 13 mitochondrial protein-coding genes of 11,349 nucleotides. Branch lengths and topology are from the BI analysis. Numbers above branches specify posterior probabilities from Bayesian inference (BI) and bootstrap percentages from maximum likelihood (ML, 1000 replications) analyses. The GenBank numbers for the mitochondrial genomes of all species are also shown.

## Nucleotide sequence accession numbers

The complete mitochondrial genomes of *Callospermophilus lateralis* and *Urocitellus richardsonii* (Rodentia: Sciuridae) have been assigned GenBank accession numbers KP698975 and KP698976, respectively.

## References

[CIT0001] Castresana J 2000 Selection of conserved blocks from multiple alignments for their use in phylogenetic analysis. Mol Biol Evol. 17:540–552. 10742046 10.1093/oxfordjournals.molbev.a026334

[CIT0002] Harrison RG, Bogdanowicz SM, Hoffmann RS, Yensen E, Sherman PW 2003 Phylogeny and evolutionary history of the ground squirrels (Rodentia: Marmotinae). J Mamm Evol. 10:249–276.

[CIT0003] Helgen KM, Cole FR, Helgen LE, Wilson DE 2009 Generic revision in the Holarctic ground squirrel genus *Spermophilus*. J Mammal. 90:270–305.

[CIT0004] Herron MD, Castoe TA, Parkinson CL 2004 Sciurid phylogeny and the paraphyly of Holarctic ground squirrels (*Spermophilus*). Mol Phylogenet Evol. 31:1015–1030. 15120398 10.1016/j.ympev.2003.09.015

[CIT0005] Huelsenbeck JP, Ronquist F 2001 MRBAYES: Bayesian inference of phylogenetic trees. Bioinformatics. 17:754–755. 11524383 10.1093/bioinformatics/17.8.754

[CIT0006] Li BF, Yu DN, Cheng HY, Storey KB, Zhang JY 2015 The complete mitochondrial genomes of *Cynomys leucurus* and *C. ludovicianus* (Rodentia: Sciuridae). Mitochondrial DNA. [Epub ahead of print]. doi: 10.3109/19401736.2015.1015010. 25693710

[CIT0007] Swofford DL 2002 PAUP*: phylogenetic analysis using parsimony (*and Other Methods). Version 4.0b10. Sunderland (MA): Sinauer Associates.

[CIT0008] Zhang LP, Storey KB, Yu DN, Hu Y, Zhang JY 2015 The complete mitochondrial genome of *Ictidomys tridecemlineatus* (Rodentia: Sciuridae). Mitochondrial DNA. [Epub ahead of print]. doi:10.3109/19401736.2015.1041117. 26024127

